# Supplementary material for: Modeling glioblastoma heterogeneity as a dynamic network of cell states
Source: Mol Syst Biol. 2021 Sep 16;17(9):e10105. doi: 10.15252/msb.202010105 (PMC8444284; doi:10.15252/msb.202010105)
Supplement: Supplementary file 6 — Source Data for Figure 5 [file MSB-17-e10105-s004.zip › Figure5A_sourcedata/GSEA_3071/hallmarks_stateII.GseaPreranked.1623742536692/HALLMARK_IL2_STAT5_SIGNALING.html]

Details for gene set HALLMARK\_IL2\_STAT5\_SIGNALING[GSEA]

|  || Dataset | state23071 |
| Phenotype | NoPhenotypeAvailable |
| Upregulated in class | na\_pos |
| GeneSet | HALLMARK\_IL2\_STAT5\_SIGNALING |
| Enrichment Score (ES) | 0.29467395 |
| Normalized Enrichment Score (NES) | 1.6187855 |
| Nominal p-value | 0.011709602 |
| FDR q-value | 0.04536084 |
| FWER p-Value | 0.329 |
Table: GSEA Results Summary

  

Fig 1: Enrichment plot: HALLMARK\_IL2\_STAT5\_SIGNALING      
 Profile of the Running ES Score & Positions of GeneSet Members on the Rank Ordered List

  

| PROBE | GENE SYMBOL | GENE\_TITLE | RANK IN GENE LIST | RANK METRIC SCORE | RUNNING ES | CORE ENRICHMENT || 1 | CDC6 |  |  | 11 | 1.631 | 0.0419 | Yes |
| 2 | LIF |  |  | 64 | 1.184 | 0.0614 | Yes |
| 3 | GADD45B |  |  | 146 | 0.965 | 0.0676 | Yes |
| 4 | PIM1 |  |  | 184 | 0.896 | 0.0830 | Yes |
| 5 | ALCAM |  |  | 288 | 0.771 | 0.0785 | Yes |
| 6 | AHNAK |  |  | 355 | 0.708 | 0.0815 | Yes |
| 7 | KLF6 |  |  | 357 | 0.708 | 0.1006 | Yes |
| 8 | UCK2 |  |  | 374 | 0.695 | 0.1156 | Yes |
| 9 | CCND3 |  |  | 390 | 0.683 | 0.1306 | Yes |
| 10 | LRRC8C |  |  | 419 | 0.669 | 0.1419 | Yes |
| 11 | ODC1 |  |  | 430 | 0.664 | 0.1576 | Yes |
| 12 | PNP |  |  | 434 | 0.659 | 0.1749 | Yes |
| 13 | SLC39A8 |  |  | 474 | 0.640 | 0.1827 | Yes |
| 14 | NT5E |  |  | 481 | 0.637 | 0.1986 | Yes |
| 15 | MYO1C |  |  | 490 | 0.632 | 0.2139 | Yes |
| 16 | SOCS1 |  |  | 593 | 0.581 | 0.2045 | Yes |
| 17 | BCL2L1 |  |  | 687 | 0.536 | 0.1961 | Yes |
| 18 | PHTF2 |  |  | 707 | 0.530 | 0.2059 | Yes |
| 19 | SLC1A5 |  |  | 757 | 0.514 | 0.2078 | Yes |
| 20 | HOPX |  |  | 768 | 0.510 | 0.2192 | Yes |
| 21 | MYC |  |  | 790 | 0.502 | 0.2277 | Yes |
| 22 | PLEC |  |  | 796 | 0.500 | 0.2402 | Yes |
| 23 | BCL2 |  |  | 813 | 0.494 | 0.2497 | Yes |
| 24 | RHOB |  |  | 881 | 0.476 | 0.2461 | Yes |
| 25 | PLAGL1 |  |  | 892 | 0.473 | 0.2565 | Yes |
| 26 | LCLAT1 |  |  | 947 | 0.460 | 0.2557 | Yes |
| 27 | PUS1 |  |  | 961 | 0.455 | 0.2649 | Yes |
| 28 | NOP2 |  |  | 983 | 0.448 | 0.2720 | Yes |
| 29 | MAFF |  |  | 986 | 0.447 | 0.2837 | Yes |
| 30 | PLSCR1 |  |  | 992 | 0.446 | 0.2947 | Yes |
| 31 | CCNE1 |  |  | 1045 | 0.429 | 0.2935 | No |
| 32 | NRP1 |  |  | 1372 | 0.362 | 0.2225 | No |
| 33 | AHCY |  |  | 1471 | 0.344 | 0.2076 | No |
| 34 | SCN9A |  |  | 1489 | 0.342 | 0.2128 | No |
| 35 | COL6A1 |  |  | 1633 | 0.319 | 0.1860 | No |
| 36 | ENPP1 |  |  | 1681 | 0.313 | 0.1829 | No |
| 37 | SWAP70 |  |  | 1751 | 0.304 | 0.1741 | No |
| 38 | CD83 |  |  | 1838 | 0.294 | 0.1608 | No |
| 39 | UMPS |  |  | 1923 | 0.282 | 0.1477 | No |
| 40 | EMP1 |  |  | 1987 | 0.274 | 0.1396 | No |
| 41 | XBP1 |  |  | 1995 | 0.273 | 0.1453 | No |
| 42 | CAPG |  |  | 2029 | 0.269 | 0.1445 | No |
| 43 | NFIL3 |  |  | 2056 | 0.267 | 0.1453 | No |
| 44 | P2RX4 |  |  | 2101 | 0.262 | 0.1416 | No |
| 45 | CKAP4 |  |  | 2131 | 0.259 | 0.1415 | No |
| 46 | ARL4A |  |  | 2167 | 0.255 | 0.1398 | No |
| 47 | POU2F1 |  |  | 2340 | -0.264 | 0.1043 | No |
| 48 | RORA |  |  | 2513 | -0.287 | 0.0695 | No |
| 49 | WLS |  |  | 2563 | -0.295 | 0.0654 | No |
| 50 | IGF1R |  |  | 2700 | -0.316 | 0.0404 | No |
| 51 | PLPP1 |  |  | 2728 | -0.322 | 0.0425 | No |
| 52 | IGF2R |  |  | 2736 | -0.324 | 0.0496 | No |
| 53 | PRAF2 |  |  | 2918 | -0.359 | 0.0145 | No |
| 54 | HIPK2 |  |  | 3029 | -0.379 | -0.0024 | No |
| 55 | CD81 |  |  | 3196 | -0.427 | -0.0319 | No |
| 56 | SPRY4 |  |  | 3201 | -0.429 | -0.0212 | No |
| 57 | ITGA6 |  |  | 3315 | -0.461 | -0.0366 | No |
| 58 | PRNP |  |  | 3383 | -0.491 | -0.0398 | No |
| 59 | IFITM3 |  |  | 3578 | -0.589 | -0.0718 | No |
| 60 | PHLDA1 |  |  | 3622 | -0.617 | -0.0656 | No |
| 61 | GABARAPL1 |  |  | 3667 | -0.657 | -0.0585 | No |
| 62 | TNFRSF21 |  |  | 3669 | -0.660 | -0.0407 | No |
| 63 | BMPR2 |  |  | 3715 | -0.698 | -0.0328 | No |
| 64 | CDKN1C |  |  | 3821 | -0.809 | -0.0367 | No |
| 65 | GPX4 |  |  | 3915 | -0.986 | -0.0328 | No |
| 66 | RGS16 |  |  | 3996 | -1.227 | -0.0191 | No |
| 67 | SYT11 |  |  | 4047 | -1.617 | 0.0126 | No |
Table: GSEA details [plain text format]

  

Fig 2: HALLMARK\_IL2\_STAT5\_SIGNALING: Random ES distribution      
 Gene set null distribution of ES for **HALLMARK\_IL2\_STAT5\_SIGNALING**
